# Headache in Post-COVID-19 Patients: Its Characteristics and Relationship with the Quality of Life

**DOI:** 10.3390/medicina58101500

**Published:** 2022-10-21

**Authors:** Endang Mutiawati, Hendrix Indra Kusuma, Marhami Fahriani, Harapan Harapan, Syahrul Syahrul, Nasrul Musadir

**Affiliations:** 1 Department of Neurology, School of Medicine, Universitas Syiah Kuala, Banda Aceh 23111, Aceh, Indonesia; 2 Department of Neurology, Dr. Zainoel Abidin Hospital, Banda Aceh 23126, Aceh, Indonesia; 3 Medical Research Unit, School of Medicine, Universitas Syiah Kuala, Banda Aceh 23111, Aceh, Indonesia; 4 Faculty of Mathematics and Natural Sciences, Universitas Syiah Kuala, Banda Aceh 23111, Aceh, Indonesia; 5 Faculty of Tarbiyah and Teacher Training, Universitas Islam Negeri Ar-Raniry, Banda Aceh 23111, Aceh, Indonesia; 6 Department of Microbiology, School of Medicine, Universitas Syiah Kuala, Banda Aceh 23111, Aceh, Indonesia; 7 Tropical Disease Centre, School of Medicine, Universitas Syiah Kuala, Banda Aceh 23111, Aceh, Indonesia

**Keywords:** post-COVID-19 headache, prolonged COVID-19, COVID-19 sequelae, long COVID

## Abstract

Little is known on the characteristics of headaches associated with coronavirus disease 2019 (COVID-19) in Indonesia. The objective of this study was to describe the characteristics of headache in post-COVID-19 patients, and its impact on the patients’ quality of life (QoL), as well as to determine the associated determinants of the poor QoL. A cross-sectional study was conducted in Banda Aceh, Indonesia. The demographic characteristics, clinical symptoms of COVID-19, characteristics of headache, and the QoL were collected and assessed. Headache was diagnosed and characterized using the International Classification of Headache Disorders, version 3 (ICHD-3). QoL was assessed using a Short Form 36 Health Survey (SF-36) tool. A logistic regression model was used to investigate the associated determinants of poor QoL in post-COVID-19 patients. A total of 215 post-COVID-19 patients were included in the final analysis, and 21.4% (46/215) of them had a poor QoL due to headache following COVID-19. Those who were unemployed and who contracted COVID-19 less than three months prior to the study had higher odds of having poor QoL compared to those who were employed and who contracted COVID-19 more than three months prior to the study. Low QoL was also related to headache that occurred less than one month after recovering from COVID-19 (compared to that which occurred longer than one month after); had a high frequency; had a combination sensation of pulsating, pressing, fiery, and stabbing pain; had a high severity score; and had additional symptoms accompanying the headache. In conclusion, headache related to COVID-19 is associated with low QoL among post-COVID-19 patients. A guideline on prevention measures of headache on COVID-19 patients, therefore, needs to be established to avoid long-term consequences.

## 1. Introduction

In late 2019, coronavirus disease 2019 (COVID-19), caused by severe acute respiratory syndrome coronavirus 2 (SARS-CoV-2), emerged, and confirmed cases rapidly increased worldwide, which led to a global pandemic in March 2020 [1,2,3]. Since then, every country has faced the consequences, not only from the infection itself, but also the economics, border restrictions, and disruptions of public services [4,5,6]. Although effective vaccines are available, high vaccine hesitancy is reported globally [7,8]. Most SARS-CoV-2 infections are asymptomatic; however, in symptomatic patients, complaints range from fever, cough, and dyspnea to more severe symptoms [9,10,11,12,13,14,15,16,17]. SARS-CoV-2 infection not only affects the respiratory system, but can also have neurological manifestations, such as dizziness, ageusia, headache, and muscle pain [10,18,19,20]. Persistent COVID-19 symptoms have also been reported following COVID-19 recovery worldwide [21].

COVID-19 affects people from all backgrounds, ethnicities, and age groups [22,23,24,25,26]. Factors affecting the quality of life (QoL) of COVID-19 patients have previously been reported [27]. However, the QoL of patients with persistent symptoms after COVID-19 recovery is still scarce. Persistent COVID-19 not only affects the respiratory system, but also the neurological, gastrointestinal, and psychosocial systems of patients [28,29,30]. Distress and sleeping disorders were found to be the most common persistent psychological symptoms after COVID-19 recovery (36%; 95%CI: 22–51% and 35%; 95%CI: 29–41%, respectively) [30]. A follow-up study showed that hospitalized COVID-19 survivors showed neurocognitive impairment, psychiatric morbidity, and reduced QoL after two months [31]. Headache is the most common symptom after 6 and 9 months of COVID-19 recovery, with a prevalence of 47.1% [32,33,34]. However, the QoL in COVID-19 survivors with prolonged headache is not clearly understood. Patients with persistent headache often complain of bilateral headaches with a pressing sensation, with varying degrees of severity from mild to severe [35,36]. The vast majority of COVID-19 patients, 72%, reported that the post-COVID-19 headaches were different during the acute phase of COVID-19 [37]. The characteristics of headaches among COVID-19 survivors in Indonesia are unavailable. Therefore, this study was conducted: (a) to determine the characteristics of headache in post-COVID-19 patients in Indonesia; (b) to assess the impacts of headache on the QoL of post-COVID-19 patients; and (c) to determine the associated determinants of poor QoL of post-COVID-19 patients.

## 2. Materials and Methods

### 2.1. Study Design and Eligibility Criteria

A cross-sectional study was conducted to assess the characteristics of persistent headaches in post-COVID-19 patients and their relationship to QoL in a provincial referral hospital in Aceh, Indonesia. The study was conducted at the Neurology Polyclinic of Dr. Zainoel Abidin Hospital in Banda Aceh, the capital of Aceh. Patients who seek treatment at Dr. Zainoel Abidin Hospital come from all regions of Aceh, and, therefore, it could provide an overview of the characteristics of patients who represent Aceh.

All patients over 18 years who were confirmed to have COVID-19 by polymerase chain reaction (PCR) and who had a headache during the course of COVID-19 or who developed a headache after being discharged from the hospital were considered eligible. Patients were excluded if they were diagnosed with other secondary headaches prior to COVID-19.

### 2.2. Study Variables

The International Classification of Headache Disorders version 3 (ICHD-3) was used to collect the headache characteristics and to make a headache diagnosis [36]. Headache characteristics included data on the first time the headache occurred (divided into: during acute COVID-19, 1–4 weeks, 1–3 months, 4–6 months, >12 months after recovering from COVID-19), whether they had to take an analgesic when the headache occurred, the location of the headache, the duration of the headache attack (divided into <1, 1–6, 7–12, >12 h a day), the frequency (1–2 times/month, 1–2 times/week, >2 times per week or everyday), the sensation (pulsating, pressing, fiery, stabbing, or combination), the severity of the headache, and additional symptoms accompanying the headache.

The primary response variable of this study was the QoL of patients with persistent headache after COVID-19 recovery. The QoL was measured using a 36-item Short Form Health Survey (SF-36) [37]. This questionnaire consists of 36 questions that are divided into 8 health concepts: physical function, pain, role limitations due to physical problems, role limitations due to personal or emotional problems, mental health, social function, vitality, and general health perceptions. Physical function was assessed by using ten questions about how the participant’s health limit their activities, such as vigorous activities (running or lifting heavy objects), moderate activities (moving a table or pushing a vacuum cleaner), climbing one or several flights of stairs, walking short (one block) or long distances (more than a mile). Bodily pain was evaluated by the intensity of pain described by the participants during the past four weeks, and how the pain interferes with their daily activities. Four questions were used to assess how the participants’ physical health limits their work or daily activities, including whether they accomplished less than before, have difficulty performing work, or if they have cut down the time spent working with a yes or no response. The impact of the participants’ emotional state on work or daily activities was evaluated by three questions: whether they accomplished less than before, were careless, or spent less time working. SF-36 also includes two questions about how much of the time and to what extent the physical health or emotional problems affect the participants’ social function. The participants’ vitality was evaluated by four questions about whether they feel tired or worn out or the opposite, and whether they feel they have a lot of energy. Five questions were used to address their emotional well-being, which included whether they are a nervous or happy person, and whether they feel calm or down. Finally, the participants’ general health was examined by questions such as if they seem to get sick easily, or expect their health to get worse, or even the opposite, if they feel their health is excellent. The participants were asked to choose a response to constitute a hierarchical Guttman scale in physical function questions, in which the score for each item consistently decreases in severity or difficulty, as follows: yes, limited a lot (1); yes, limited a little (2); or no, not limited at all (3). The other questions are rated on Likert-type or frequency response scales, ranging from three response categories for the physical function items to six categories for the bodily pain items. Using the standard guideline, the scores were transformed into a linear 0 to 100 range, with a higher score representing a better QoL [38].

Explanatory variables assessed in this study included demographic characteristics, COVID-19 vaccination, the presence of specific symptoms of COVID-19, and headache characteristics. Sociodemographic data consisted of age (divided into 20–29, 30–39, 40–49, and >50-year-old), gender, occupation (grouped into employed and unemployed), monthly income (divided into <3, 3–5, 5–10, and >10 million Indonesian rupiah), and COVID-19 vaccination status (had dose 1, dose 2, or booster shot), time of COVID-19 infection (<3, 3–6, 7–12, and >12 months ago), and whether they were hospitalized during the last COVID-19 course. The presence of the specific symptoms of COVID-19, such as fever, shortness of breath, cough, cold, headache, muscle pain (myalgia), anosmia, ageusia, diarrhea, or nausea/vomiting, were also assessed, as well as the presence of comorbidities, such as hypertension, diabetes, obesity, kidney illness, heart disease, lung disease, liver disease, tumor or cancer, autoimmune disease, asthma, or others.

### 2.3. Statistical Analysis

The SF-36 score was used to determine the frequency (percentage) of patients’ QoL categories in eight domains, and the results were classified as good or poor QoL based on a cut-off point of 50%. A logistic regression model was used to investigate the relationships between the explanatory variables and QoL of the patients. IBM SPSS Statistics version 22 was used, and *p* < 0.05 was considered statistically significant.

### 2.4. Ethics Approval and Patient Consent

The Ethics Committee of Dr. Zainoel Abidin Hospital approved the study protocol under the number 010/EA/FK-RSUDZA/2022. Following human research regulations, all patients provided written informed consent before participating this study.

## 3. Results

### 3.1. Respondents’ Characteristics

We included 215 post-COVID-19 patients in the final analysis. The socio-demographic characteristics of the respondents are presented in Table 1. Before contracting COVID-19, more than half (62.8%) of the respondents were unvaccinated against the disease. Of those vaccinated, 37.2% received the first dose, and 39.1% were fully vaccinated. Almost half of the respondents (45.6%) were aged between 30–39 years old, and 25.6% were between 20–29 years old. Females were more dominant than males (69.8 vs. 30.2%). About 65% of the participants were working, and 52.1% earned IDR 3–5 million. More than a third (34.4%) of the respondents contracted COVID-19 less than 3 months prior to the study. The vast majority of the respondents (90.2%) were not hospitalized during COVID-19 infection. Of those hospitalized (9.8%), only 0.9% were admitted to the ICU or RICU. Most participants complained of fever (77.2%) and headache (72.1%), whereas anosmia and ageusia only presented in 45.1% and 32.6%, respectively. A total of 20% of the participants had at least one underlying condition, such as hypertension (7.0%), obesity (5.6%), diabetes (5.1%), asthma (4.2%), cardiovascular disease (0.9%), autoimmune disease (1.4%), or kidney diseases (0.5%).

### 3.2. Headache Characteristics

Most participants (155/215, 72.1%) experienced headache during hospitalization or self-isolation for COVID-19, with 71.6% of them taking medication to relieve the pain (Table 2). Headache was felt all over the head by 47.4% (102/215) of the participants, whereas 21.4% of the participants could not describe the location of the pain. Among the respondents, 36 (16.7%) indicated that their headache only occurred in one area (frontoparietal, 5.6%; frontal, 5.1%; and parietal, 4.2%). Headache occurred for less than one hour in 133/215 (61.9%) of the respondents, whereas 37.7% experienced the headache for longer. Headache was complained about everyday by 33.0% of the participants, whereas for 83 of 215 (38.6%) participants, only once or twice per month. More than half of the participants (127/215, 59.1%) described the headache as a pulsating pain, 20.9% as pressing pain, 6.0% as mixed between pulsating and pressing pain, and 5.1% as stabbing pain. Based on the severity of the headache, moderate headache (46.5%), mild headache (42.3%), and severe headache (11.1%) were experienced by the participants. Some factors, including physical activities, noise, and bright light, or their combination, aggravated the headache, whereas rest, pain medication, and a combination of rest and pain medication helped ease the pain.

Ninety-five of the participants (44.2%) had additional symptom occurrence with the headache, including cough (31.6%), myalgia (29.8%), fever (29.3%), nausea/vomiting (17.2%), and anosmia/ageusia (12.6%).

### 3.3. QoL of the Participants with Headache in COVID-19 Infection

The QoL of the participants are presented in Table 3. The majority of the participants (80.9%) had good physical functioning; however, 36.7% had limitations due to physical problems, and 34.4% due to emotional problems (Table 3). There were 3.3% participants with poor mental health due to the headache, 17.7% with fatigue, 38.6% with pain, and 21.9% with poor social function that interferes with their daily life, meaning 56/215 (26.0%) of the participants reported poor general health. Overall, 46/215 (21.4%) of the participants had a poor QoL due to headache following COVID-19 infection.

### 3.4. Factors Associated with the QoL Based on the Participants’ Characteristics

Our study showed that unemployment status and contracting COVID-19 less than 3 months prior to the study were associated with poor QoL (*p* < 0.05). Individuals with headache who had received the first and second dose of the COVID-19 vaccine were approximately 2.5 times more likely to have poor QoL compared to those who were unvaccinated (OR: 0.39; 95%CI: 0.18–0.84 and OR: 0.41; 95%CI: 0.20–0.87, respectively). Participants who had been COVID-19-positive less than 3 months prior to the study had almost five times greater odds of poor QoL compared to those who were infected between 7–12 months prior to the study (OR: 0.19, 95%CI: 0.06–0.59, with *p* = 0.004) and those who were positive more than 12 months prior to the study (OR:0.21, 95%CI: 0.08–0.52, with *p* = 0.001). Age group, sex, the history of hospitalization, monthly income, symptoms during COVID-19 infection, and comorbidity were not associated with the QoL of the participants (Table 4).

### 3.5. Factors Associated with the QoL Based on the Headache Characteristics

The headache characteristics that are associated with the QoL of the participants are presented in Table 5. The participants who started to have headache 1–4 weeks after recovering from COVID-19 had approximately nine times greater odds of poor QoL compared to those who had headache after one month from COVID-19 recovery, with OR: 8.85; 95%CI: 1.05–74.50, and *p* = 0.027. It is understandable that poor QoL was associated with those who took the medication (OR: 7.49, 95%CI: 2.23–25.18). Having a headache between 1–6 h or more than 6 h were associated with increased odds of poor QoL compared to participants who experienced the headache for less than 1 h (OR: 2.35, 95%CI: 1.16–4.77, and OR: 4.24, 95%CI: 1.33–13.52, respectively). Experiencing headache everyday was associated with nine times greater odds of poor QoL compared to those who only had headache once-to-twice per month (OR: 9.13, 95%CI:3.33–24.98, *p* = 0.001).

Having a combination of a pulsating, pressing, and stabbing headache increased the odds of poor QoL by almost five times compared to a headache with a pulsating sensation only (OR: 4.77, 95%CI: 2.00–11.41, *p* < 0.001). Having a severe headache also increased the odds of poor QoL by almost eight times compared to participants who only had a mild headache, with OR: 7.71, 95%CI: 2.68–22.20, and *p* < 0.001. Participants with additional symptoms accompanying headaches were more than four times as likely to have poor QoL (OR: 4.38, 95%CI: 2.15–8.95). The location of the headache and the factors that aggravate and ease the headache were not associated with the QoL of the participants (Table 5).

## 4. Discussion

Headache is one of the main neurological symptoms of SARS-CoV-2 infection, and is almost two times more prevalent in COVID-19 patients than other respiratory viral infections [38]. A previous study showed that the global prevalence of headache in COVID-19 was 25.2% (26,464 out of 104,751 cases) [39]. Headache was the first reported symptom of COVID-19 in 42.2% (84/199), with prolonged headache persisting in 13.6% (27/199), and of those, more than 10.2% (20/199) lasted for more than 3 months [38]. The proposed pathomechanism of headache in COVID-19 patients is probably the result of direct neuronal damage of the trigeminal nerve by SARS-CoV-2, or the indirect effects of hypoxia, coagulopathy, and cytokine storm (such IL-1β, IL-6, and TNF-α) that are involved in various pathological pain mechanisms [40,41].

The phenotype of headache in this study was bilateral (47.4%) with a pulsating sensation (59.1%), which required 71.6% of the participants to take medication which relieved the pain. Similar results were found in a previous study showing that pulsating headache (50.9%) was predominant in COVID-19 patients with prior headache, and the majority of the patients also needed to take analgesics (84.3%); of those, analgesics improved the pain in only 32% cases, and 12.4% completely recovered from headache [42]. The phenotype of headache in COVID-19 infection shows similarity with migraine or tension-type headache, and should be cautiously analyzed [43]. Headache in other viral infections, such as dengue, also occurred bilaterally; however, these were mostly reported in a throbbing (59.2%) or pressing pattern (40.7%), and were usually associated with nausea, photophobia, and phonophobia [44,45].

The majority of the participants (80.9%) in this study had good physical functioning, with only 36.7% having limitations due to physical problems, and 34.4% due to emotional problems.

Overall, more than a fifth of the participants had a poor QoL due to headache following COVID-19 infection related to pain, social function, fatigue, and mental health. A recent study found that post-COVID-19 sequalae affected the ability to perform self-care, increased anxiety/depression disorder, and reduced usual activities such as full-time employment [46]. Cognitive dysfunction and reduced usual activities and self-care highlight that patients with post-COVID-19 sequelae may have a reduced ability to participate in social functions [46]. The poor QoL in COVID-19 survivors with headache was associated with unemployment, as well as contracting COVID-19 less than 3 months prior the study. Economic downturns, such as difficulty in obtaining food and medicine, temporary or permanent layoffs, and a decrease in monthly income, might increase the financial burden, which results in an impaired QoL in patients with post-COVID-19 syndrome. A significant improvement in work productivity, self-reported good health, functional status, and health-related QoL were also reported between 3-6 months after COVID-19 recovery [47].

Our study also found that participants with a combination of pulsating, pressing, and stabbing headache were 4.7 more likely to have a poor QoL, and when they had to take painkillers, this increased to 7.5 greater odds of an impaired QoL. A previous study found that participants with migraine-like symptoms, such as pulsating headache, aggravation by physical activity, photophobia, and phonophobia, have a higher headache intensity and self-rated disability [48]. The pooled prevalence of poor QoL in COVID-19 survivors with persistent headache was 21% (95%CI: 3–47%) based on the EQ-5D-5L questionnaire [49]. Another study found that a prior history of migraines worsens the QoL in COVID-19 survivors with headache as a sequelae, with a Headache Impact Test-6 (HIT-6) score of 58.6 ± 13.2 when compared to no prior migraine (47 ± 12.2, *p* = 0.005) [50]. These results suggest that migraine-like symptoms in long COVID-19 headache are concerning due to their association with the course of the disease and their effect in the patients’ quality of life [48]. Being partially or non-responsive to analgesic treatment is the characteristic of headache in COVID-19 patients, which is why previously, indomethacin has been proposed as an alternative choice of drug [51]. To shed light in differentiating prolonged headache in COVID-19 patients from other headaches, several criteria were set for the ICHD-3 as a guideline for physicians and researchers to validate future investigations [52].

There are some limitations of this study that should be discussed. The number of the sample size is relatively low. Therefore, further studies that include a bigger sample size from multiple centers from the Indonesian archipelago would provide more comprehensive results. In our present study, the QoL of the individuals was assessed using the SF-36 questionnaire. Although this tool is valid and commonly used in population studies, it is less commonly used to assess the QoL in headache patients. Therefore, further studies might also need to include a more specific questionnaire for headache, such as the Headache Attributed Lost Time (HALT). In addition, the QoL tool from WHO [53] could also be considered to enhance the comparability between studies around the globe.

## 5. Conclusions

Investigating the phenotypic criteria of COVID-19-related headache is important, as the impact of this symptom is debilitating in patients’ quality of life. Physicians and researchers should not only differentiate the characteristics of headache in COVID-19 to other diseases, but also unravel the mysteries of the triggering mechanism of headache in COVID-19; thus, a better understanding of the management of COVID-19-related headache might be achieved.

## Figures and Tables

**Table 1:** Characteristics of the respondents included in this study (n = 215).

Characteristics	n	%
Age group (year)		
20–29	55	25.6
30–39	98	45.6
40–49	41	19.1
>50	21	9.8
Gender		
Male	65	30.2
Female	150	69.8
Employment		
Unemployed	76	35.3
Employed	139	64.7
Monthly income (Indonesian Rupiah)		
<3 million	46	21.4
3–5 million	112	52.1
5–10 million	45	20.9
>10 million	12	5.6
Time between COVID-19 and the study		
<3 months (*R*)	74	34.4
3–6 months	29	13.5
7–12 months	43	20.0
>12 months	69	32.1
Hospitalization for COVID-19		
No	194	90.2
Yes	21	9.8
Admitted to the intensive unit (ICU) or RICU due to COVID-19		
No	213	99.1
Yes	2	0.9
COVID-19 symptom		
Fever	166	77.2
Shortness of breath	31	14.4
Cough and cold	146	67.9
Headache	155	72.1
Muscle pain/myalgia	111	51.6
Anosmia	97	45.1
Ageusia	70	32.6
Diarrhea	26	12.1
Nausea/vomiting	42	19.5
Asymptomatic	8	3.7
Comorbidity		
Hypertension	15	7.0
Diabetes	11	5.1
Obesity	12	5.6
Kidney illness	1	0.5
Heart disease	2	0.9
Lung disease	0	0.0
liver disease	0	0.0
Tumor/cancer	0	0.0
Autoimmune disease	3	1.4
Asthma	9	4.2
Others	6	2.8
None	172	80.0

**Table 2:** Characteristics of headache reported by post-COVID-19 patients in Indonesia (n = 215).

Characteristics	n	%
Time between COVID-19 and the headache		
During treatment/during self-isolation	155	72.1
1–4 weeks after recovered	36	16.7
1–3 months after recovered	9	4.2
4–6 months after recovered	3	1.4
>12 months after recovered	5	2.3
Unable to remember	7	3.3
Had painkillers for the headache		
No	61	28.4
Yes	154	71.6
Location of the headache		
Only in one point/area	36	16.7
Right side	20	9.3
Left side	11	5.1
Whole head	102	47.4
Cannot be described	46	21.4
Duration of the headache		
Less than 1 h	133	61.9
1–6 h	68	31.6
7–12 h	4	1.9
More than 12 h	10	4.6
Frequency of the headache		
1–2 times/month	83	38.6
1–2 times/week	32	14.9
>2 times/week	16	7.4
Everyday	84	39.1
Characteristic of the headache		
Pulsating	127	59.1
Pressing	45	20.9
Fiery	4	1.9
Stabbing	11	5.1
Combination pulsating and pressing	13	6.0
Combination pulsating and stabbing	4	1.9
Combination pressing and fiery	1	0.5
Combination pressing and stabbing	2	0.9
Combination pulsating, pressing, and stabbing	2	0.9
Combination pulsating, fiery, and stabbing	6	2.8
Severity of headache (on a pain scale of 0–10, where 0 is no pain and 10 is very painful)		
0	3	1.4
1–3	88	40.9
4–6	100	46.5
7–9	22	10.2
10	1	0.9
What makes the headache worse		
Activity/tired	97	45.1
Bright light	10	4.7
Noise	15	7.0
Activity/tired and bright light	4	1.9
Activity/tired and noise	20	9.3
Bright light and noise	4	1.9
All three	9	4.2
None	56	26.0
What makes the headache better		
Rest	76	35.3
Painkiller	43	20.0
Rest and painkiller	73	33.5
None	23	10.7
Additional symptom during headache		
No	111	55.8
Yes	95	44.2
Type of symptom during headache		
Fever	63	29.3
Shortness of breath	22	10.2
Cough and cold	68	31.6
Myalgia	64	29.8
Anosmia/ageusia	27	12.6
Diarrhea	10	4.7
Nausea/vomiting	37	17.2
Photophobia	8	3.7
Phonophobia	4	1.9

**Table 3:** Quality of life of respondents based on the domains (n = 215).

QoL Domain	Mean ± SD	Category	
		Good n (%)	Poor n (%)
Physical functioning	674.66 ± 224.85	174 (80.9)	41 (19.1)
Role limitations due to physical health	206.06 ± 139.79	136 (63.3)	79 (36.7)
Role limitations due to emotional problems	186.51 ± 103.35	141 (65.6)	74 (34.4)
Pain	112.16 ± 41.12	132 (61.4)	83 (38.6)
Energy/fatigue	245.26 ± 63.62	177 (82.3)	38 (17.7)
Emotional well-being	365.07 ± 70.08	208 (96.7)	7 (3.3)
Social functioning	121.26 ± 38.65	168 (78.1)	47 (21.9)
General health	290.44 ± 79.88	159 (74.0)	56 (26.0)
Total QoL	2201.41 ± 517.85	169 (78.6)	46 (21.4)

**Table 4:** Sociodemographic and COVID-19 factors associated with the QoL of patients.

Factor	n	%	Poor QoL n (%)	OR (95%CI)	*p*-Value
Age group (year)					
20–29 (*reference group* (*R*))	55	25.6	14 (25.5)	1	
30–39	98	45.6	14 (14.3)	0.49 (0.21–1.12)	0.090
40–49	41	19.1	9 (22.0)	0.82 (0.32–2.14)	0.691
>50	21	9.8	9 (42.9)	2.20 (0.76–6.31)	0.144
Sex					
Male (*R*)	65	30.2	10 (15.4)	1	
Female	150	69.8	36 (24.0)	1.74 (0.80–3.76)	0.160
Employment					
Unemployed (*R*)	47	21.9	17 (36.2)	1	
Employed	168	78.1	29 (17.3)	0.37 (0.18–0.75)	0.006
Monthly income (Indonesian Rupiah)					
<3 million (*R*)	46	21.4	9 (19.6)	1	
3–5 million	112	52.1	25 (22.3)	1.18 (0.50–2.77)	0.702
5–10 million	45	20.9	11 (24.4)	1.33 (0.49–3.60)	0.575
>10 million	12	5.6	1 (8.3)	0.37 (0.04–3.28)	0.375
Time between COVID-19 and the study					
<3 months (*R*)	74	34.4	26 (35.1)	1	
3–6 months	29	13.5	9 (31.0)	0.83 (0.33–2.09)	0.693
7–12 months	43	20.0	4 (9.3)	0.19 (0.06–0.59)	0.004
>12 months	69	32.1	7 (10.1)	0.21 (0.08–0.52)	0.001
Hospitalization for COVID-19					
No (*R*)	194	90.2	39 (20.1)	1	
Yes	21	9.8	7 (33.3)	1.99 (0.75–5.26)	0.167
Admitted to the intensive unit (ICU) or RICU due to COVID-19					
No (*R*)	213	99.1	46 (21.6)	1	
Yes	2	0.9	0 (0.0)	0.00 (0.00–NA)	0.999
Additional symptoms when positive for COVID-19					
Headache	163	75.8	39 (23.9)	2.02 (0.84–4.85)	0.114
Others (*R*)	52	24.2	7 (13.5)	1	
Loss of smell (anosmia)	97	45.1	20 (20.6)	0.92 (0.48–1.77)	0.801
Others (*R*)	118	54.9	26 (22.0)	1	
Loss of taste (ageusia)	70	32.6	14 (20.0)	0.88 (0.44–1.79)	0.729
Others (*R*)	145	67.4	32 (22.1)	1	
Comorbidity					
No comorbidity (*R*)	172	80.0	36 (20.9)	1	
Had comorbidity	43	20.0	10 (23.3)	1.15 (0.52–2.54)	0.740

**Table 5:** Headache characteristics factors associated with the QoL.

Factor	n	%	Poor QoL n (%)	OR (95%CI)	*p*-Value
Time between COVID-19 and the headache					
During treatment/during self-isolation	155	72.1	35 (22.6)	6.71 (0.88–51.45)	0.067
1–4 weeks after recovered	36	16.7	10 (27.8)	8.85 (1.05–74.50)	0.027
More than 1 month (*reference group* (*R*))	24	11.2	1 (4.2)	1	
Had painkillers for the headache					
No (*R*)	61	28.4	3 (4.9)	1	
Yes	154	71.6	43 (27.9)	7.49 (2.23–25.18)	0.001
Location of the headache					
Only in one point/area (*R*)	36	16.7	4 (1.1)	1	
Right side	20	9.3	1 (5.0)	0.42 (0.04–4.05)	0.454
Left side	11	5.1	1 (9.1)	0.80 (0.08–8.01)	0.849
Whole head and indescribable	148	68.8	40 (27.0)	2.96 (0.99–8.91)	0.053
Duration of the headache					
<1 h (*R*)	133	61.9	20 (15.0)	1	
1–6 h	68	31.6	20 (29.4)	2.35 (1.16–4.77)	0.017
>6 h	14	6.5	6 (42.9)	4.24 (1.33–13.52)	0.015
Frequency of the headache					
1–2 times/month	83	38.6	5 (6.0)	1	
1–2 times/week	32	14.9	7 (21.9)	4.37 (1.27–14.99)	0.019
>2 times/week	16	7.4	3 (18.8)	3.60 (0.77–16.91)	0.105
Every day and NA	84	39.1	31 (36.9)	9.13 (3.33–24.98)	<0.001
Characteristic of the headache					
Pulsating (*R*)	127	59.1	22 (17.3)	1	
Pressing	45	20.9	7 (15.6)	0.88 (0.35–2.22)	0.786
Fiery	4	1.9	0 (0.0)	0.00 (0.00–NA)	0.999
Stabbing	11	5.1	3 (27.3)	1.79 (0.44–7.29)	0.417
Any combination of those	28	13.0	14 (50.0)	4.77 (2.00–11.41)	<0.001
Severity score of headache					
0–3 (*R*)	91	42.3	9 (9.9)	1	
4–6	100	46.5	26 (26.0)	3.20 (1.41–7.27)	0.005
>6	24	11.2	11 (45.8)	7.71 (2.68–22.20)	<0.001
What makes the headache worse					
None (*R*)	56	26.0	11 (19.6)	1	
Activity/tired	97	45.1	20 (20.6)	1.06 (0.47–2.42)	0.885
Bright light	10	4.7	1 (10.0)	0.46 (0.05–3.98)	0.476
Noise	15	7.0	2 (13.3)	0.63 (0.12–3.21)	0.577
Combination	37	17.2	12 (32.4)	1.96 (0.76–5.09)	0.165
What makes the headache better					
None (*R*)	23	10.7	3 (13.0)	1	
Rest	76	35.3	10 (13.2)	1.01 (0.25–4.03)	0.989
Painkiller	43	20.0	14 (32.6)	3.22 (0.82–12.68)	0.095
Rest and painkiller	73	34.0	19 (26.0)	2.35 (0.63–8.79)	0.206
Having additional symptoms during headache					
No (*R*)	120	55.8	13 (10.8)	1	
Yes	95	44.2	33 (34.7)	4.38 (2.15–8.95)	<0.001

## Data Availability

The data of the study can be obtained from the corresponding author with an acceptable reason.
